# Correlation between reproductive hormones levels and semen quality in patients with diabetes

**DOI:** 10.25122/jml-2022-0079

**Published:** 2022-12

**Authors:** Baida Rihan Ali, Ahmed Nasir Alameri, Shaymaa AL Rumaidh, Saleem Ethaib

**Affiliations:** 1 Department of Pathological Analysis, College of Science, University of Thi-Qar, Al-Nasiriya, Iraq; 2 Department of Physiology, College of Medicine, University of Thi-Qar, Al-Nasiriya, Iraq; 3 Department of Environmental Engineering, College of Engineering, University of Thi-Qar, Al-Nasiriya, Iraq

**Keywords:** diabetes mellitus, male infertility, hormones, semen analysis

## Abstract

Diabetes mellitus (DM) is rarely sought among infertile patients due to a lack of studies and inconsistency regarding its impact on semen quality. This cross-sectional study aimed to determine the influence of T2DM on the pituitary gland hormones (FSH and LH) in males. A total of 60 participants participated in this study, of which 35 were diagnosed with diabetes mellitus, and 25 were without diabetes. Fasting blood sugar, HbA1c, LH, FSH, TT, E2, and prolactin were tested. Diabetic men had lower serum LH, FSH, and TT levels than non-diabetics and higher prolactin and E2 levels. According to the semen examination, including sperm count, PH, motility, and morphology, diabetic patients had considerably lower sperm counts, motility, and morphology than non-diabetic patients. In conclusion, the decrease in the concentration of reproductive hormones in diabetic patients leads to sexual weakness, resulting in abnormal seminal fluid parameters, which are below the normal levels than in apparently healthy persons.

## INTRODUCTION

Diabetes mellitus (DM) is one of the most important public health problems facing society today, and the prevalence of the disease is rapidly increasing. According to the World Health Organization (WHO), there were around 171 million people with diabetes in 2000, showing a 60% increase over the preceding decade. Type 2 diabetes mellitus (T2DM) is a major public health concern in the world today [1]. Southeast Asia has the world's second-highest number of diabetes, behind Africa. Males of Southeast Asian ancestry have a sixfold greater prevalence of type 2 diabetes than other males [2]. In addition to impairing a range of physiological systems, T2DM can also damage the reproductive system. Furthermore, it can generate autonomic neuropathy and endothelial dysfunction in the body and cause male sexual dysfunction.

According to Gandhi J et al. (2017) [3], subfertility is a serious health and social problem that affects around 10% of the world's population. A more in-depth examination of fertility rates in modern culture reveals that the growing incidence of diabetes is significantly linked to diminishing birth and fertility rates in modern nations [4]. This has occurred as a result of an alarming increase in the number of diabetic men of reproductive age. A growing number of patients with diabetes mellitus (DM) have been recorded among men of reproductive age, and the incidence of DM is strongly related to fertility loss [5]. The glucose levels in the blood influence and modify the function of many organs and tissues. Blood glucose levels are carefully controlled in the liver and fat, which is especially essential because these play crucial roles in nutrition utilization and storage via hormonally regulated systems.

Furthermore, glucose metabolism is an important stage in the testing procedure and is necessary for the *in vivo* maintenance of spermatogenesis [6]. According to a study, insulin levels in the blood affect the sperm plasma membrane and acrosome. As a result, spermatogenesis is hindered in people with diabetes with insulin resistance or insufficiency [7]. Diabetes can negatively impact erectile and ejaculatory function, as well as decrease sperm volume, sperm counts, sperm motility, and result in abnormal sperm morphology [8]. Given that the global diabetes burden is continually increasing, with an estimated prevalence of 422 million people in 2014, the number of men of reproductive age who have diabetes will also increase [9, 10]. Therefore, this work aimed to examine the relationship between type 2 diabetes mellitus, sperm parameters (sperm count, semen volume, motility, and morphology), and sex hormones in infertile men at the time of the study.

## MATERIAL AND METHODS

Blood samples were collected from patients at Al-Hussein Teaching Hospital and Ibn Al-Bitar laboratory in Thi-Qar province, Iraq, between 1/8/2020 and 1/11/2020. This study included 25 healthy male participants aged 30 to 59 years and 35 people with diabetes. Participants were divided into three groups, according to age: the first group (control) with ages between 30–59 years, the second group: diabetic patients between 30–45 years, and the third group: diabetic patients between 36– 59 years. Sperm and blood samples were collected between 9:00 A.M. and 10:00 A.M. The blood samples were divided into two groups of 5 ml each. First, 1 ml of blood was drawn and placed in EDTA tubes and tested for hemoglobin (Hb) and other hematological parameters using an automatic hematological assay analyzer (Nihon Kohden Corporation, Japan). Then 4 ml of blood was drawn and placed in plain centrifuge tubes at room temperature to allow the blood to clot. The serum was separated by centrifugation at 3000 g for 30 minutes, and fasting blood glucose (FBG), follicle-stimulating hormone (FSH), luteinizing hormone (LH), total testosterone (TT), estradiol (E2), and prolactin (PRL) hormones were determined in the serum. Seminal fluid samples were also obtained in a separate room near the laboratory where they were tested. Sperm samples were taken after three to seven days of abstention from sexual activity. All samples were obtained by masturbation and ejaculation into a container made of glass or plastic from a batch proven non-toxic to spermatozoa. The samples were brought to the laboratory in an average of 10 minutes and promptly placed in an incubator where they were permitted to completely liquefy.

### Statistical analysis

The statistical analysis was carried out using (SPSS) version 24. We used descriptive statistics such as frequencies, relative frequencies, means, and standard deviations. In addition, the Chi-Square test, simple correlation (r), and simple linear regression were used to assess the associations between parameters. Results were considered statistically significant at the probability level of P≤0.05.

## RESULTS

The results revealed a statistically significant increase (P=0.05) in the HbA1c level. When the third group was compared to the control and second group, the results revealed a statistically significant increase in HbA1c level. However, when the second group was compared to the control group, the findings demonstrated a statistically significant decrease in HbA1c level (Table 1).

**Table 1:** FBG, HbA1c in control and patients with T2DM (according to age).

Groups	FBG mg/dl	HbA1c %
Parameters		
**First group (control) 30–59 years**	96.24±1.10 ^b^	5.01±0.12 ^c^
**Second group 30–45 years**	280.77±8.17 ^a^	9.72±1.00 ^b^
**Third group 46–59 years**	265.13±5.22 ^a^	11.00±1.23 ^a^
**LSD**	56.78	1.30

Values are means±S.E.; The difference in letters is significant (p≤0.05). The same letters denote non-significant differences (p≤0.05); LSD – Least significant difference

When comparing the second and third groups with the first (control) group, the results revealed a significant decrease (P=0.05) in FSH, LH, and TT and a significant increase (P=0.05) in E2 and PRL in the second and third groups (Table 2).

**Table 2:** Reproductive hormone in control and patients with T2DM (according to age).

Groups	FSHmlU/ml	LHmlU/ml	TTng/mI	E2Pg/ml	PRLng/ml
Parameters					
**First group (control) 30–59 year**	7.05±1.22 ^a^	4.13±0.87 ^a^	4.22±0.77 ^a^	21.81±1.06 ^b^	9.11±0.99 ^b^
**Second group 30–45 years**	3.12±0.24 ^b^	2.82±0.51 ^b^	2.80±0.67 ^b^	32.63±1.27 ^a^	13.89±1.12 ^a^
**Third group 46–59 years**	2.76±0.52 ^b^	2.57±0.41 ^b^	2.32±0.38 ^b^	30.23±0.87 ^a^	19.27±1.33 ^a^
LSD	1.11	0.91	0.57	7.52	3.28

Values are expressed in means±S.E.; The difference in letters is significant (p≤0.05). The same letters denote non-significant differences (p≤0.05); LSD – Least significant difference

The results showed a significant decrease (P≤0.05) in PH in patient groups compared to the control group. Also, there was a significant increase in liquefaction and motility (sluggish and dead) sperm in the patient groups compared to the control group. There was a significantly increased sperm morphology (abnormal sperm) in the second group compared with the third and the control groups, and a significantly increased sperm morphology (abnorma sperm) in the third group compared with the control group (Table 3).

**Table 3:** Results of semen analysis in control and patients with T2DM.

Groups		First group (control) 30–59 years	Second group 30–45 years	Third group 46–59 years	LSD
Parameters					
**PH**		7.91±0.12 ^a^	7.18±0.09 ^b^	7.11±0.40 ^b^	0.15
**Volume (ml)**		3.72±0.31	3.45±0.29	3.35±0.28	NS
**Liquefaction (min)**		28.42±0.76 ^b^	32.12±2.18 ^a^	31.32±0.95 ^a^	3.82
**Viscosity**		1.00±0.05	1.07±0.05	1.08±0.06	NS
**Concentration (×106/ml)**		65.54±3.81 ^a^	26.83±3.67 ^b^	33.45±3.39 ^b^	9.97
**Motility %**	Progressive (A)	33.35±1.56 ^a^	7.21±0.87 ^b^	5.72±0.80 ^b^	4.67
	Non-Progressive (B)	35.88±2.00 ^a^	13.58±1.11 ^b^	15.23±1.81 ^b^	4.96
	Sluggish (C)	14.27±0.68 ^b^	32.55±3.04 ^a^	31.15±2.91 ^a^	7.98
	Dead (D)	20.00±2.01 ^b^	50.13±9.24 ^a^	48.09±6.21 ^a^	12.27
**Morphology %**	Normal	87.11±2.53 ^a^	45.01±4.66 ^b^	57.13±5.21 ^b^	13.77
	Abnormal	16.01±0.98 ^c^	57.00±3.87 ^a^	39.76±2.21 ^b^	14.11

Values are expressed in mean±S.E; The difference in letters is significant (p≤0.05). The same letters denote non-significant differences (p≤0.05); NS – represents non-significant between groups.

## DISCUSSION

Among the subjects investigated, the fasting blood sugar (FBS) level ranged from 280.77 mg/dl to 265.13 mg/dl, with a mean value of 8.175.22 mg/d. The hemoglobin A1c (HbA1c) test is a critical tool for evaluating glycemic control and has a high predictive value for diabetic complications [11]. There was a statistically significant increase in HbA1c levels in diabetic patients (p<0.05). This demonstrates that HbA1c levels are significantly connected with abnormal blood glucose levels in diabetic individuals, which is supported by other studies [12]. According to the patient's medical history, we found that the HbA1c level of diabetic patients was in an average steady state over the previous three months, with no short-term variations. As a result, the risk of significant consequences from diabetes can be reduced by monitoring and regulating blood sugar levels throughout time. According to Heiskanen (2013), diabetes control, as indicated by HbA1c, may be affected by age distribution, with younger people having better control than older people [13, 14]. Diabetic complications were more common among the elderly due to uncontrolled diabetes, which puts them at greater risk of developing complications. In 2009, Mirzazadeh et al. [14] published a paper about the factors related to the disparity of diabetes care in Iran and found that the age distribution of the sample was responsible for the variance in the appearance of diabetic complications. T2DM can also cause damage to organs. Diabetes is associated with a number of chronic consequences, including hypogonadism, nonalcoholic fatty liver disease, osteoporosis, cancer, and other diseases. Hypogonadism has a negative impact on the quality of life of diabetic individuals. According to this study, hypogonadotropic (low LH and FSH) gonadal dysfunction was the most prevalent type in diabetic individuals. When comparing diabetes patients of both sexes to healthy persons, Hussein and Al-Qatsi (2012) [15] discovered a statistically significant decrease in serum LH and FSH. A study published in 1993 found that people with diabetes had significantly greater FSH and LH levels than non-diabetics. Because insulin has no stimulatory effect on Leydig cells and because FSH levels decreased and resulted in lower LH levels, Leydig cell activity and testosterone production are lower in T2DM patients. Rendong et al. (2016) [16] investigated the link between TT, LH, and FSH and discovered that the low testosterone groups had lower levels of LH and FSH and that testosterone was positively linked with both LH and FSH levels. Clinical studies revealed that 25 percent of people with T2DM also had low levels of LH and FSH, in addition to low testosterone [17].

Subnormal free testosterone concentrations in males with T2DM were originally observed in combination with abnormally low luteinizing hormone (LH) and follicle-stimulating hormone concentrations [18]. The intensity of hyperglycemia did not affect the appearance of these anomalies. The incidence of hypogonadotropic hypogonadism (HH) in males with type 2 diabetes has been found at 30–40%. In a study published in 2008 [19], younger men with T2DM had a comparable high prevalence of hypogonadotropic hypogonadism (HH). It was unexpected to see a lack of significant mean difference in LH and FSH between the groups. This might be due to the high prevalence of primary hypogonadism, which is related to testicular problems rather than gonadotropins [20]. This result agrees with another study [21] which reported that TT in males is principally synthesized in the Leydig cells, and LH and FSH control the number of Leydig cells. In addition, the amount of TT generated by the present Leydig cells is under the control of LH, which regulates the expression of 17-B hydroxyl steroid dehydrogenase. When comparing the results of this investigation to the control, the E2 level increased considerably (P>0.05). This might be due to the enzyme aromatase, which transformed TT to E2 in the first place. This finding is consistent with a research conducted in Baghdad in 2016 on T2DM males between the ages of 37 and 66 years old, which found that elevated E2 levels in T2DM were caused by the enzyme aromatase, which is related to obesity, and associated with T2DM [22].

In the presence of the aromatase enzyme, which is present in adipose tissue, TT is converted to E2, which may be the most prevalent cause of decreased TT concentration in people with diabetes and obese individuals. Kelly and Jones (2013) found that testosterone has beneficial effects on several cardiovascular risk factors, which include cholesterol, endothelial dysfunction, and inflammation [23]. Arnold et al. (2010) [24] showed a substantial increase in serum prolactin in diabetes patients compared to controls, while Daimon et al. (2017) [25] found a significant increase in serum prolactin in diabetic patients compared to controls, which contradicts our findings. Increased prolactin levels in patients with type 2 diabetes may also serve as a compensatory mechanism against hyperglycemia, as prolactin is essential for enhancing pancreatic-cell function and overcoming insulin resistance in this condition. This was confirmed in a study conducted by Ruiz-Herrera et al., who discovered that the administration of prolactin via osmotic mini-pumps into rodent adipose tissue improves insulin sensitivity, increases the expression of GLUT4, reduces the expression of inflammatory cytokines in visceral fat, and prevents adipocyte hypertrophy [26]. When comparing the diabetes group to the non-diabetic group, this study found a statistically significant decrease in the mean of semen parameters (PH, concentration, motility, and morphology of sperm), increasing interstitial collagen, seminiferous tubule thickness, peritubular and intertubular fibrosis, or gonadal problems caused by diabetes' inadequate circulation to the testis.

According to earlier research, patients with type 2 diabetes have been reported to have a much higher frequency of decreased sperm motility and morphology [27, 28]. These findings are consistent with those of Delfino et al., who discovered that patients with diabetes had significantly lower rates of normal sperm morphology and motility than non-diabetic patients in their study [29]. In light of the aberrant sperm morphology and diabetes, it is evident that sperm cells, as rapidly growing cells, are particularly sensitive to the oxidative stress generated by both conditions [30].

## CONCLUSION

The decreases in the concentration of reproductive hormones in diabetic patients lead to sexual weakness, which leads to abnormal seminal fluid parameters below the normal levels than in apparently healthy persons.

## Personal thanks

The authors would like to acknowledge the Department of Pathological Analysis, College of Science, University of Thi-Qar, for their assistance, advice, and technical support. We also thank the patients who agreed to use part of their samples to support the present study.
